# Pulmonary benign metastasizing leiomyoma: a case report and review of the literature

**DOI:** 10.1186/1477-7819-10-268

**Published:** 2012-12-12

**Authors:** Yili Fu, Hui Li, Bo Tian, Bin Hu

**Affiliations:** 1 Department of Thoracic Surgery, Beijing Chao-Yang Hospital, Capital Medical University, Beijing, 100020, People’s Republic of China

**Keywords:** Lung, Leiomyoma, Benign lesion, Metastases

## Abstract

Pulmonary benign metastasizing leiomyoma characterized by the growth of uterine leiomyoma in the lung is a very rare disease. We herein report the case of a 46-year-old asymptomatic woman who underwent a total abdominal hysterectomy for her multiple uterine leiomyomas 5 years ago, with the presence of multiple shadows in her chest roentgenogram during the regular check-up. Chest computerized tomography (CT) showed multiple solitary nodules in both lungs. Video-assisted thoracoscopic surgery with a wedge resection of the lesion was performed. Histopathologically, the pulmonary nodule was composed of benign smooth muscle cells and demonstrated low mitotic activity and absence of necrosis. Immunohistochemical staining for smooth muscle actin (SMA) and Desmin were extremely positive. CD10, CD117 and S-100 were negative in the tumor cells. Positive immunoreactivity for estrogen receptor (ER) and progesterone receptor (PR) were detected. The pathological diagnosis was pulmonary benign metastasizing leiomyoma.

## Background

Pulmonary benign metastasizing leiomyoma is a very rare disease characterized by the growth of uterine leiomyoma tissue in the lung [1]. In most cases, there is a previous history of total abdominal hysterectomy for uterine leiomyoma. However, the pathogenesis of this disease has not yet been elucidated.

## Case presentation

**Figure 1:**
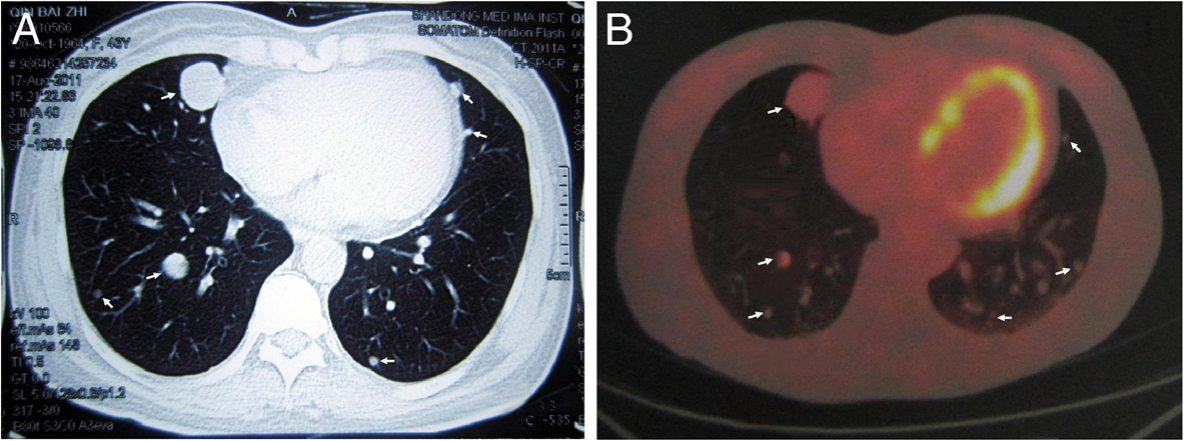
**A Chest computerized tomography scan shows multiple nodules (arrows) in both lungs. B** The PET scan shows no abnormal fluorodeoxyglucose uptake with suspicion of multiple nodules, and the SUV value was about 2.

A 46-year-old Chinese woman, who was a nonsmoker, was admitted for further examination of scattered shadows in both lungs that were found incidentally on the chest roentgenogram during a regular checkup in 2011. A chest computerized tomography (CT) was subsequently performed. The patient’s past medical history showed a diagnosis of multiple uterine leiomyoma in 2005 for which a total abdominal hysterectomy had been performed. The pathology revealed a uterine smooth muscle tumor with vascular invasion. During the 5-year follow-up, no recurrence of the benign metastasizing leiomyoma was detected. The clinical examination was unremarkable, and the results from routine laboratory examinations were normal. CT demonstrated multiple solitary nodules in both lungs with maximum diameters of 2 cm (Figure 1A). Positron emission tomography (PET/CT) did not show abnormal fluorodeoxyglucose uptake with the suspicion of multiple nodules, and the SUV value was about 2 (Figure 1B). The diagnosis at the time of admission was suspected pulmonary metastasizing leiomyoma.

**Figure 2:**
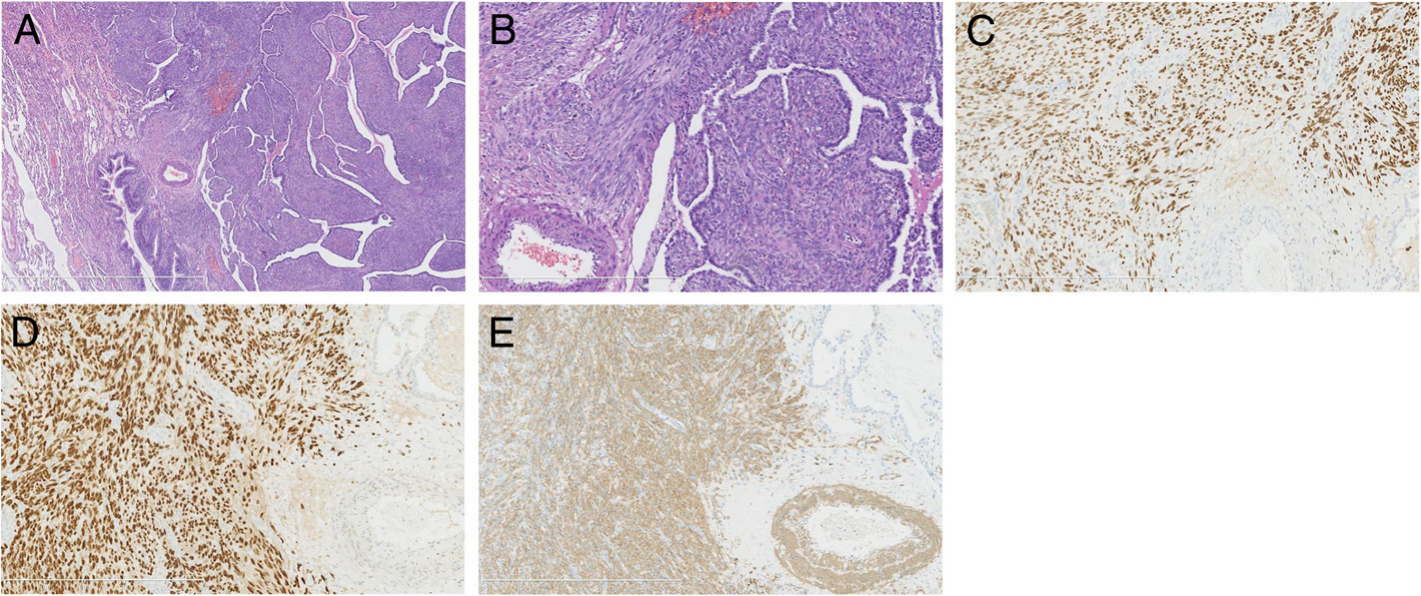
**A Pathological examination of the nodule in the right lung reveals that the tumor is composed of spindle-shaped muscle cells and distinct cell borders (hematoxylin and eosin staining, ×2.5). B** Pathological examination demonstrated low mitotic activity, little cytological cellular atypia and no tumor cell necrosis (hematoxylin and eosin staining, **×**10). **C** Immunohistochemical staining reveals positive smooth muscle actin (SMA). CD10, CD117 and S-100 were negative in the tumor cells. Positive immunoreactivity for estrogen receptor (ER). **D** Immunohistochemical staining shows positive estrogen receptor of pulmonary nodules, and the progesterone receptor (PR) suggested that the spindle-shaped cells were uterine smooth muscle cells. **E** Immunohistochemical staining for smooth muscle actin (SMA) and desmin was strongly positive, suggesting that the spindle-shaped cells were smooth muscle cells.

In August 2011, the patient underwent right video-assisted thoracoscopic surgery with wedge resection of the lesion. One of the lesions from the right lower lobe was resected for pathological diagnosis. There were multiple small nodules in the right lower lobe, which were purplish red, soft and smooth, with clear demarcations. Intraoperative pathological examination (frozen pathology) revealed a benign leiomyoma. Postoperative pathological examination showed a pulmonary benign metastasizing leiomyoma, consisting of revealed spindle-shaped cells with abundant clear cytoplasm and distinct cell borders. It demonstrated low mitotic activity (<0-1/10 high-power fields), little cytological cellular atypia and no tumor cell necrosis (Figure 2A). Immunohistochemical staining showed positive progesterone receptors of pulmonary nodules (Figure 2B-2E). The Ki67 index was 5%.

The patient was discharged from the hospital without any early postoperative complications. She started taking mifepristone (12.5 mg daily) and receiving Zoladex injections (3.6 mg every 4 weeks). Her current general condition was satisfactory without any radiological evidence of recurrent disease or distant metastases. The remaining lesions in both lungs were stable during the follow-up period.

## Discussion

Benign metastasizing leiomyoma is a very rare disease in which tissue from a benign uterine leiomyoma is detected as a solitary nodule or as multiple nodules in the lungs of patients with a previous history of hysterectomy for uterine leiomyoma. In 1939, Steiner et al. reported the first case in which a leiomyoma transferred from the uterus to the lung, showing a benign histological appearance. It was called “metastasizing fibroleiomyoma” [1]. Since then there have been several similar reports, and it was renamed benign metastasizing leiomyoma.

Most patients with benign metastasizing leiomyoma are asymptomatic. It is usually found during physical examinations. Abramson et al. reported that the average age of patients with benign metastasizing leiomyoma is 48 years old, with the period from hysterectomy to nodule detection varying from 3 months to 26 years [2]. Horstmann et al. reported that the radiological presentation of pulmonary benign metastasizing leiomyoma is as multiple nodules in 87% of cases (70% bilateral nodules and 17% unilateral nodules) or as a solitary nodule in 13% of cases [3]. The main metastatic site of benign metastasizing leiomyoma is the lung, but other sites including the lymph nodes, soft tissue of the pelvis, bone, bone marrow, greater omentum, peritoneum and heart have also been reported [4]. Tsunoda et al. reported the first case of benign metastasizing leiomyoma of the lung complicated with primary lung cancer [5].

The nature and etiology of benign metastasizing leiomyoma are still controversial. Uterine leiomyoma has been reported to depend on hormones [1,6]. Estrogen receptor and progesterone receptor in case of benign metastasizing leiomyoma usually have high expression, and drugs or ovariectomy can make the lesions regress or remain stabilized. This provides us with a basis for believing that estrogen and progesterone may play an important role in the course of pathogenesis. However, Patton et al. have previously reported that benign metastasizing leiomyoma results from the monoclonal, hematogenous spread of an apparently benign uterine leiomyoma, most likely leading to secondary intravenous leiomyomatosis [7].

Making the diagnosis of benign metastasizing leiomyoma is very difficult, and it has strict pathological diagnostic criteria. It is based on the results of the immunohistochemical staining and the history of hysterectomy for uterine leiomyoma. In our case the pathological examination showed spindle-shaped cells without mitotic activity or nuclear atypia. The tumor cells are strongly positive for SMA, confirming the smooth muscle origin of this benign tumor. In addition to these features, immunohistochemical staining is negative for CD117, ruling out an extra-gastrointestinal stromal tumor, and all of the cells are negative for S100, essentially excluding a tumor of neural origin. Positive immunoreactivity for estrogen receptor (ER) and progesterone receptor (PR) suggests the spindle-shaped cells are uterine smooth muscle cells.

There are currently no standard treatment guidelines for benign metastasizing leiomyoma. Reported treatment modalities include careful observation, surgical resection, hysterectomy and bilateral oophorectomy, progestins, aromatase inhibitor and medical castration using luteinizing hormone-releasing hormone analogs [8] (Table 1). However, surgery combined with hormone therapy is believed to be the best choice of treatment for benign metastasizing leiomyoma [9]. A radical surgical resection has been advocated as the primary treatment for pulmonary benign metastasizing leiomyoma if possible. The use of long-acting GnRH analogs, which suppress the endogenous gonadotropin secretion necessary for gonadal steroid production, has been suggested as the best option for unresectable cases. The role of surgery for those unresectable cases is to provide a precise histological diagnosis.

**Table 1:** Review of the current literature

**Author**	**Publication dates**	**Case no.**	**Treatment**	**Current status**
Goto [10]	2012	1	Refused hormone therapy after surgery	Stable
Taveira-DaSilva [11]	2012	1	Treatment with leuprolide acetate	Improved
Ahmad [12]	2011	1	Monthly injections of depot GnRH analogs	Stable
Lim [13]	2011	1	Observation	Stable
Hara [14]	2011	1	Observation	Stable
Ogawa [15]	2011	1	Observation	Stable
Yoon [9]	2011	1	Taking an aromatase inhibitor (Letrozole® 2.5 mg daily, Femara)	Stable
Rivera [4]	2004	2	Patient 1.Taking the long-acting GnRH agonist, leuprolide acetate. Five months later, an AI at a dose of 1 mg daily was added. Refused the addition of an LHRH agonist	Stable
			Patient 2. On follow-up a month after discharge, the patient was convinced to restart raloxifene 120 mg daily and anastrozole 2 mg daily	
Kayser [16]	2000	10	Treatment with cytostatic in 2 patients, with hormonal therapy in 2 and observation in 6	Stable

## Conclusions

Although BML is a very rare condition, it should be considered in asymptomatic women of reproductive age with a history of uterine leiomyoma who present with solitary or multiple pulmonary nodules of the lung. Long-term close surveillance is required for patients with BML for early detection of recurrence or distant metastases.

### Consent

Written consent was obtained from the patient for the study and publication of this case report and accompanying images. A copy of written consent is available for review from the Editor-in-Chief.

## Competing interests

The authors declare that they have no competing interests.

## Authors’ contributions

YF collected information and prepared the original draft. HL researched the relevant literature and revised the draft. BT helped with the literature research and preparing the manuscript. BH helped prepare the manuscript. All authors read and approved the final manuscript.
